# SAMHD1-induced endosomal FAK signaling promotes human renal clear cell carcinoma metastasis by activating Rac1-mediated lamellipodia protrusion

**DOI:** 10.1038/s12276-023-00961-x

**Published:** 2023-04-03

**Authors:** Sunho An, Tam Thuy Lu Vo, Taekwon Son, Hoon Choi, Jinyoung Kim, Juyeon Lee, Byung Hoon Kim, Misun Choe, Eunyoung Ha, Young-Joon Surh, Kyu-Won Kim, Ji Hae Seo

**Affiliations:** 1 College of Pharmacy and Research Institute of Pharmaceutical Sciences, Seoul National University, Seoul, 08826 South Korea; 2 Department of Biochemistry, School of Medicine, Keimyung University, Daegu, 42601 Republic of Korea; 3 Korea Brain Bank, Korea Brain Research Institute, Daegu, 42601 Republic of Korea; 4 Department of Internal Medicine, School of Medicine, Keimyung University, Daegu, 42601 Republic of Korea; 5 Department of Urology, School of Medicine, Keimyung University, Daegu, 42601 Republic of Korea; 6 Department of Pathology, School of Medicine, Keimyung University, Daegu, 42601 Republic of Korea

**Keywords:** Lamellipodia, Endocytosis, Renal cell carcinoma, Oncogenes, RHO signalling

## Abstract

Human sterile α motif and HD domain-containing protein 1 (SAMHD1) has deoxyribonucleoside triphosphohydrolase (dNTPase) activity that allows it to defend against human immunodeficiency virus type I (HIV-1) infections and regulate the cell cycle. Although SAMHD1 mutations have been identified in various cancer types, their role in cancer is unclear. Here, we aimed to investigate the oncogenic role of SAMHD1 in human clear cell renal cell carcinoma (ccRCC), particularly as a core molecule promoting cancer cell migration. We found that SAMHD1 participated in endocytosis and lamellipodia formation. Mechanistically, SAMHD1 contributed to the formation of the endosomal complex by binding to cortactin. Thereafter, SAMHD1-stimulated endosomal focal adhesion kinase (FAK) signaling activated Rac1, which promoted lamellipodia formation on the plasma membrane and enhanced the motility of ccRCC cells. Finally, we observed a strong correlation between SAMHD1 expression and the activation of FAK and cortactin in tumor tissues obtained from patients with ccRCC. In brief, these findings reveal that SAMHD1 is an oncogene that plays a pivotal role in ccRCC cell migration through the endosomal FAK-Rac1 signaling pathway.

## Kidney cancer: Protein involved in cancer spread explored

A protein that is overexpressed in kidney cancer drives metastasis by regulating a specific pathway, and provides a potential therapeutic target. Sunho An at Seoul National University, South Korea, and co-workers examined the role of the SAMHD1 protein in the promotion and metastasis of renal clear cell carcinoma. SAMHD1 mutations are found in multiple cancers and have been linked to resistance to chemotherapy. The team analysed extensive datasets and found that high levels of SAMHD1 expression are associated with poor prognoses. Using human cell cultures, they showed that the protein is involved in processes that facilitate the migration and invasion of cancer cells. For example, SAMHD1 binds to another protein and promotes the formation of lamellipodia, protrusions on the leading edge of the cancer cell membrane that aid migration, by inducing a particular signaling pathway.

## Introduction

Human sterile α motif and HD domain-containing protein 1 (SAMHD1) was originally identified as a protein whose mutation is related to Aicardi–Goutières syndrome, which is an autoimmune disease^1^. Later, SAMHD1 was discovered to act as a deoxyribonucleoside triphosphohydrolase (dNTPase) in mammals. The most well-studied biological functions of SAMHD1 are involved in the early immune response against viruses, such as human immunodeficiency virus type I (HIV-I), hepatitis B virus, and herpes simplex virus 1. Previous studies revealed that SAMHD1 hydrolyzes intracellular dNTPs—essential components for viral reverse transcription or DNA replication—at the early stage of viral infection^1–4^. Since several previous studies have observed SAMHD1 nuclear accumulation^1,5,6^, SAMHD1 was presumed to function in the nucleus. However, inactivation of the nuclear localization sequence (NLS) affects neither the dNTPase nor the restriction activities of SAMHD1, demonstrating that nuclear localization is not a prerequisite for these functions^7,8^. Cytoplasmic SAMHD1 is protected from Vpx-mediated proteasomal degradation and can prevent HIV-I infection^5,8^. A previous study reported that SAMHD1 is translocated from the nucleus to the cytoplasm through growth factor stimulation^9^, suggesting that SAMHD1 may perform different functions depending on its intracellular location.

Recently, SAMHD1 was reported to be associated with cancer progression. In total, 1 542 SAMHD1 mutations have been identified in various cancer types, including chronic lymphocytic leukemia, mantle cell lymphoma, and colorectal cancer^10–12^. SAMHD1 promotes chemoresistance because, given the structural similarity to endogenous dNTP, the nucleotide analogs used for acute myeloid leukemia and mantle cell lymphoma treatments are degraded by SAMHD1^13–15^. In contrast, cell proliferation in lung adenocarcinoma (LUAD) is suppressed by SAMHD1 overexpression^16^. Therefore, the roles of SAMHD1 in cancer are unclear and controversial. Furthermore, the role of SAMHD1 in tumorigenesis and the underlying mechanisms of SAMHD1-mediated cancer progression remain unknown.

During cell migration, the plasma membrane functions as a central site for adhesion formation, force generation, and signal transduction^17–19^. The main factors determining cell migration are membrane protrusion and focal adhesion or membrane–matrix adhesion^20^. In focal adhesion, focal adhesion kinase (FAK) is activated through autophosphorylation and forms an active FAK-Src complex^21^. The FAK-Src complex phosphorylates proteins associated with nascent adhesion and finally recruits active Rac1 to the membrane ruffles and adhesion sites^22^. Activated Rac1 accumulates cortactin at the cell periphery to form lamellipodia with wide sheet-like membrane structures. Cortactin binds to actin filaments formed on the lamellipodia to stabilize polymerized actin^23,24^. In the late adhesion stage, Rac1 activity is diminished, whereas Rho A—a member of the Rho GTPase family proteins—is activated to promote propulsion and mature focal adhesion^25,26^. Finally, cortactin phosphorylation aids the depolymerization of actin filaments on the lamellipodia so that the focal adhesions of the protruding membrane can be separated to promote cell motility^27,28^.

Sufficient biomass supplementation is important to sustain focal adhesion of the plasma membrane during cell migration. Cancer cells have adapted an endosomal trafficking system to effectively supply materials during metastasis. Endocytosis supplies important plasma membrane components of moving cells that maintain cell polarity and facilitate cell migration^29,30^. Rather than being degraded within the cell, membrane receptors are rapidly recycled from the cell membrane through endo- and exocytosis. Endocytosis proceeds with the stepwise complex formation of several proteins, including FAK and cortactin. Subsequently, Rab GTPases and early endosome antigen 1 (EEA1) wrap around the endosome–protein complex to mark the target vesicle^31,32^. The expression of this recycling system is reported to be positively correlated with cell motility and cancer metastasis^31^. Increasing evidence indicates that cell migration and endocytosis occur concomitantly; however, the precise mechanism of endocytosis activation and endosomal membrane assembly remains elusive.

This study aimed to elucidate the mechanism by which SAMHD1 regulates cell migration in clear cell renal cell carcinoma (ccRCC). We identified SAMHD1 as a new binding partner of cortactin in early endosomes and lamellipodia. Moreover, SAMHD1-mediated endosomal FAK signaling stimulated Rac1 activation to trigger lamellipodial protrusion, promoting cell motility. Collectively, our study indicated that SAMHD1 modulates ccRCC cell migration through endosomal regulation and identified SAMHD1 as a potential therapeutic target for ccRCC treatment.

## Materials and methods

### Cell culture and reagents

HK-2 (#22190, a normal kidney cell line), Caki-1 (#30046), Caki-2 (#30047), SN12C (#80025), and ACHN (#21611) cells were obtained from the Korean Cell Line Bank (Seoul, Korea). Two RCC cell lines with lower SAMHD1 expression were used for SAMHD1 overexpression experiments, and two other RCC cell lines with higher SAMHD1 expression were used for SAMHD1 knockdown experiments. HK-2 cells were grown in RPMI 1640, and the other cells were grown in Dulbecco’s modified Eagle’s medium (DMEM) with 10% fetal bovine serum (FBS) and 1% penicillin/streptomycin in a humidified atmosphere of 5% CO_2_ at 37 °C. PF573228 (#PZ0117) and fasudil (#CDS021620) were purchased from Sigma‒Aldrich (St. Louis, MO, USA). NSC23766 (#S8301), ML141 (#S7686), and Dynasore (#S8047) were obtained from Selleckchem (Houston, TX, USA).

### Transfection and stable cell line construction

SN12C and ACHN cells were transfected with siRNA targeting SAMHD1 (5′- GAUUCAUUGUGGCCAUAUA-3′) using Oligofectamine Transfection reagent (#12252011, Invitrogen, Gaithersburg, MD, USA), and Caki-2 cells were transfected with pCDNA3.1-FLAG-SAMHD1 using Lipofectamine 2000 (#11668019, Invitrogen) according to the manufacturer’s instructions.

To establish stable Caki-1 cells, pIRES-FLAG-SAMHD1 plasmids were transfected into HEK293T cells with the lentivirus packing vectors psPAX2 (Addgene, Cambridge, MA, USA) and pMD2. G (Addgene), and retroviral supernatants were harvested after 48 h. Caki-1 cells were transduced with the collected supernatants, and the transduced cells were selected for one month using blasticidin (10 μg·ml^–1^, Sigma‒Aldrich). The expression of FLAG-SAMHD1 was quantified using western blotting.

### Antibodies

An anti-SAMHD1 antibody (OTI1A1, ab128107, 1:2 000) and an anti-phosphorylated cortactin Y421 antibody (ab47768, 1:1 000) were purchased from Abcam (Cambridge, UK). Anti-E-cadherin (24E10, #3195, 1:1 000), anti-N-cadherin (13A9, #14215, 1:1 000), anti-vimentin (R28, #3932, 1:2 000), anti-snail1 (C15D3, #3879, 1:1 000), anti-ZEB1 (D80D3, #3396, 1:1 000), anti-FAK (D5O7U, #71433, 1:2 000), anti-phosphorylated FAK (Y397; D20B1, #8556, 1:1 000), and anti-EEA1 antibodies (C45B10, #3288, 1:1 000) were purchased from Cell Signaling Technology (Beverly, MA, USA). Anti-cortactin (4F11, 05-180, 1:2 000) and anti-paxillin antibodies (5H11, 05-417, 1:200) were purchased from MilliporeSigma (Burlington, MA, USA). Anti-fibronectin (F3648, 1:1 000), anti-FLAG (M2, F1804, 1:2 000), anti-β-actin (A2066, 1:2 000), and anti-α-tubulin antibodies (DM1A, T9026, 1:2 000) were purchased from Sigma‒Aldrich. For immunohistochemistry, an anti-SAMHD1 antibody (NBP1-31432, 1:50) was purchased from Novus Biologicals (Centennial, CO, USA). An anti-phosphorylated FAK (Y397) antibody (#700255, 1:50) was purchased from Invitrogen (Gaithersburg, MD, USA), and an anti-phosphorylated cortactin (Y421) antibody (LS‑C353975, 1:100) was purchased from LSBio (Seattle, WA, USA).

### The Cancer Genome Atlas (TCGA) transcriptome data analysis

From the Broad GDAC Firehose database (https://gdac.broadinstitute.org/), we downloaded RNA-sequencing (RNA-seq) and clinical data from the pankidney cohort (KIPAN), clear cell renal cell carcinoma (KIRC, ccRCC in the present study), renal papillary cell carcinoma (KIRP, PRCC in the present study), and kidney chromophobe (KICH, chRCC in the present study) datasets. In addition, we downloaded the GBMLGG, UCEC, LIHC, THCA, COADREAD, BLCA, LUAD, and LUSC datasets from TCGA. mRNA expression data were used to analyze SAMHD1 expression and epithelial–mesenchymal transition (EMT)-related genes. Gene-set enrichment analysis (GSEA) was performed using GSEA software provided by the University of California San Diego and Broad Institute^33^. Gene Ontology (GO) analysis was performed using the same software. We only considered results with a *p value* < 0.05 significant.

### Survival analysis for RCC subtypes

The RNA-Seq whole genome expression profile, corresponding clinical parameters, and follow-up information for KIRC, KIRP, and KICH were downloaded as FPKM-UQ-normalized gene expression data from the UCSC XENA database (https://xenabrowser.net/). For the analysis of *SAMHD1*, each subtype of RCC was divided into “SAMHD1-high” (top 20%) and “SAMHD1-low” (bottom 80%) mRNA expression groups. For the analysis of the 5-year survival rate, survival data for the high and low expression groups were retrieved from the UCSC Xena database and analyzed using GraphPad Prism V8.2.0 (GraphPad Software, San Diego, CA, USA). Kaplan–Meier plots were used to analyze the 5-year overall survival (OS) and progression-free survival (PFS) rates of RCC patients in TCGA. Log-rank tests for significance and Kaplan–Meier curves were generated using GraphPad Prism V8.2.0 with statistical significance set at *p* < 0.05. A univariate Cox regression analysis was performed to examine the relationship between SAMHD1 expression and OS to determine the functional significance of SAMHD1 in RCC prognosis.

### Immunoblotting and immunoprecipitation

Proteins were extracted using a lysis buffer from Cell Signaling Technology (#9803) with a protease inhibitor cocktail (Gendepot, Katy, TX, USA), and 30 μg of the lysates were used for immunoblotting. For immunoprecipitation, 900 μg protein was incubated overnight at 4 °C with the corresponding primary antibodies conjugated to G beads (MilliporeSigma) or FLAG-tagged magnetic beads (Sigma‒Aldrich). Beads were washed with a washing buffer containing 20 mM Tris (pH 8.0), 150 mM NaCl, 0.2 mM EDTA, and 0.1% Triton X-100. After SDS‒PAGE, membranes were immunoblotted using the corresponding primary antibodies and HRP-conjugated secondary antibodies. Visualization was performed using Lumi-Pico solution (Dogen, Seoul, Korea) and a LAS-4000 instrument (GE Healthcare, Chicago, IL, USA). SAMHD1 levels were quantified as integrated band intensities using ImageJ (National Institutes of Health, Bethesda, MD, USA) and normalized to the levels of the corresponding housekeeping proteins.

### Cell migration assay

Cells were plated in a 6-well plate and cultured until 100% confluency was reached. Wounds were made using a 200 μL pipette tip and washed with PBS. To reduce the influence on cell proliferation, cells were treated with 5 μM thymidine. After 24 h of incubation, the wound closure rate was measured using a phase-contrast microscope.

### Transwell migration and invasion assay

Transwell chambers (#3422, Corning, Summerville, MA, USA) were used with or without Matrigel (#354234, Corning) to perform cell migration or invasion assays. ccRCC cells (1–2.5 × 10^4^) were resuspended in serum-free medium and plated onto the inner chambers. DMEM with 10% FBS was added to the outer chambers. The cells were set aside for 24 h to allow migration and invasion before the inserts were removed and washed with PBS. Cells that migrated to or invaded the opposite side of the inserts were stained with 0.5% crystal violet; the number of cells was counted using ImageJ.

### RNA isolation and mRNA analysis

Total RNA was extracted using TRIzol reagent (Invitrogen) and reverse-transcribed into cDNA using Moloney murine leukemia virus (MMLV) reverse transcriptase (Promega, Madison, WI, USA). Quantitative real-time PCR was performed using a LightCycler 480 Instrument II (Roche, Basel, Switzerland). The comparative Ct (2^-ΔΔCt^) method was used to quantify the mRNA levels, which were normalized to the levels of the corresponding housekeeping genes.

### Immunocytochemistry

Immunocytochemistry was performed as previously described^34^. Briefly, ccRCC cells were seeded onto glass coverslips in 24-well plates (4 × 10^4^ cells per well). Cells were fixed in 4% paraformaldehyde for 15 min. After washing with PBS, cells were permeabilized in PBS containing 0.1% Triton X-100 for 10 min, blocked with 3% normal goat serum for 1 h, and then incubated with primary antibodies for 1 h. After washing with PBS, the coverslips were incubated with Alexa-488 or 546-conjugated secondary antibodies (Invitrogen) for 1 h at room temperature followed by counterstaining with 4′,6-diamidino-2-phenylindole (Sigma‒Aldrich). ProLong Gold Antifade Mountant (Invitrogen) was used for mounting, fluorescence images were obtained using confocal microscopy (LSM5 EXCITER, Carl Zeiss, AG, Germany), and immunopositive cellular structures were quantified using ImageJ.

### Immunohistochemistry

Frozen human ccRCC tissues were cut into 10 μm sections using a cryostat microtome (CM1860, Leica, Wetzlar, Germany). The sections were fixed in 100% acetone for 20 min at −20 °C and blocked with 3% goat serum for 1 h. The sections were incubated with primary antibodies overnight at 4 °C. After washing with PBS, the sections were incubated with biotin-labeled secondary antibodies (Vector Labs, Burlingame, CA, USA) and staining reactions were carried out using a VECTASTAIN Elite ABC HRP Kit (PK-6101, Vector Labs) according to the manufacturer’s instructions. The immunoreaction sections were visualized with ImmPACT DAB Substrate Peroxidase (SK-4105, Vector Labs) and counterstained with hematoxylin. Quantitative analysis of the SAMHD1-, p-Cortactin-, and p-FAK-positive areas (with brown staining) was performed with ImageJ (NIH) using the protocol provided by the LSU Health Sciences Center^35^. Images of the stained tissues were adjusted relative to the threshold using the RGB mode.

### ccRCC clinical specimen collection

We analyzed 20 pairs of human tissues (tumor and normal specimens) from Dongsan Hospital, Daegu, Korea. All specimens were obtained from patients who provided informed consent as stipulated by the Institutional Review Board at Keimyung University (School of Medicine), and the study protocol was approved by the ethics committee of Keimyung University Hospital (#2020-10-068). The tissues were subjected to western blotting and immunohistochemistry to compare SAMHD1, phospho-FAK, and phospho-cortactin levels between the matched samples.

### Statistical analysis

The results are expressed as the mean ± SD or SEM. We used a two-tailed Student’s *t test* for single comparisons or two-way ANOVA for multiple comparisons. Statistical significance was set at *p* < 0.05. GraphPad Prism V8.2.0 (GraphPad Software) was used for statistical analyses.

## Results

### High SAMHD1 expression is associated with poor prognosis in ccRCC

**Fig. 1:**
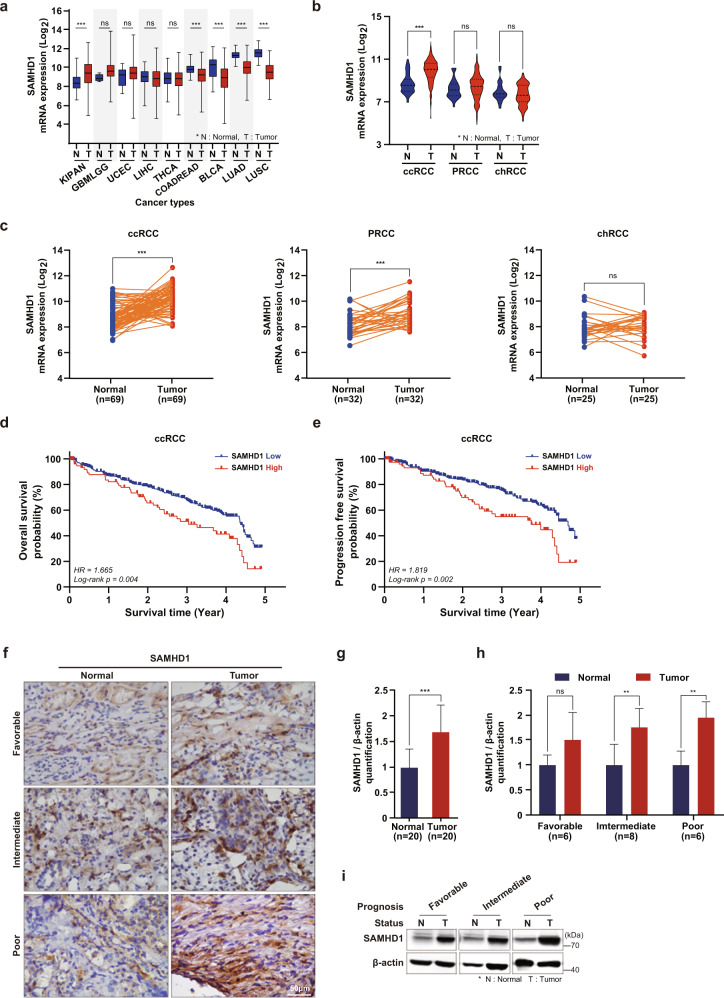
High SAMHD1 expression is associated with poor prognosis in ccRCC patients. **a** Comparing the gene expression of SAMHD1 in various cancer types using the TCGA database. KIPAN, pankidney cancer; GBMLGG, glioma; UCEC, uterine corpus endometrial carcinoma; LIHC, liver hepatocellular carcinoma; THCA, thyroid carcinoma; COADREAD, colorectal adenocarcinoma; BLCA, bladder urothelial carcinoma; LUAD, lung adenocarcinoma; LUSC, lung squamous cell carcinoma. **b** Comparison of *SAMHD1* mRNA expression between tumor and normal tissues according to histological RCC subtypes. ***p < 0.001. **c** Comparative mRNA expression of *SAMHD1* between paired tumor and nontumor tissues according to histological RCC subtypes. ***p < 0.001. **d** Kaplan–Meier analysis of 5-year overall survival (OS) between SAMHD1 high expression (*n* = 77) and SAMHD1 low expression groups (*n* = 303). Log-rank *p* = 0.004. **e** Kaplan–Meier analysis of progression-free survival (PFS) between SAMHD1 high expression (*n* = 78) and SAMHD1 low expression groups (*n* = 300). Log-rank *p* = 0.002. **f** Immunohistochemistry (IHC) of SAMHD1 in ccRCC tissues. Scale bar, 50 μm. **g** Immunoblotting was used to analyze the protein level of SAMHD1 in 20 paired cancer and normal tissues. SAMHD1 protein levels were normalized to corresponding β-actin levels. **p < 0.01. **h** SAMHD1 protein levels normalized to β-actin levels in ccRCC tissues according to patient prognosis were analyzed by western blotting. *p < 0.05. Data are presented as the mean ± SD. **i** Representative western blot bands are presented with the corresponding prognosis.

To elucidate the role of SAMHD1 in cancer, TCGA datasets were used to analyze the expression of SAMHD1 in different cancer types. Among the several types of cancer, the difference in SAMHD1 expression between cancerous and normal tissues was the highest in pankidney cancer (Fig. 1a). RCC is classified into three subtypes according to histological characteristics^36^. Accordingly, we compared *SAMHD1* mRNA levels between three RCC subtype datasets. Interestingly, only ccRCC had significantly upregulated mean *SAMHD1* mRNA expression in tumor tissues compared with normal tissues (Fig. 1b). *SAMHD1* mRNA levels were higher in tumor tissues than in adjacent normal tissues from patients with the ccRCC subtype (Fig. 1c). Furthermore, we investigated the relationship between prognosis and SAMHD1 expression in patients with ccRCC. Notably, Kaplan–Meier survival analysis revealed that patients with high SAMHD1 expression exhibited worse OS and PFS rates (Fig. 1d, e) than patients with low SAMHD1 levels. We did not observe any relationship between SAMHD1 expression and OS or PFS in PRCC and chRCC (Supplementary Fig. 1). Immunohistochemistry and western blot analysis showed higher protein expression levels of SAMHD1 in tumor tissues than in the paired normal tissues (Fig. 1f, g). Analysis of protein levels according to cancer grades indicated that SAMHD1 was overexpressed in tumor tissues with intermediate and poor prognoses. The protein level of SAMHD1 was also increased in tumor tissues with favorable prognoses compared to their adjacent normal tissues, but there was no statistically significant difference (Fig. 1h, i). Since the oncogenic effects of SAMHD1 mutations have already been reported in some cancer types, we investigated the occurrence of SAMHD1 mutations in RCC using the TCGA database. SAMHD1 mutations rarely occurred in RCC; only 4 out of 330 patients with ccRCC and 1 out of 277 patients with PRCC exhibited SAMHD1 mutations. Among the mutations, two were deletions, and three were substitutions (data not shown). Collectively, these results suggest that SAMHD1 expression may be associated with ccRCC development.

### SAMHD1 potentiates the migration and invasion of ccRCC cells

**Fig. 2:**
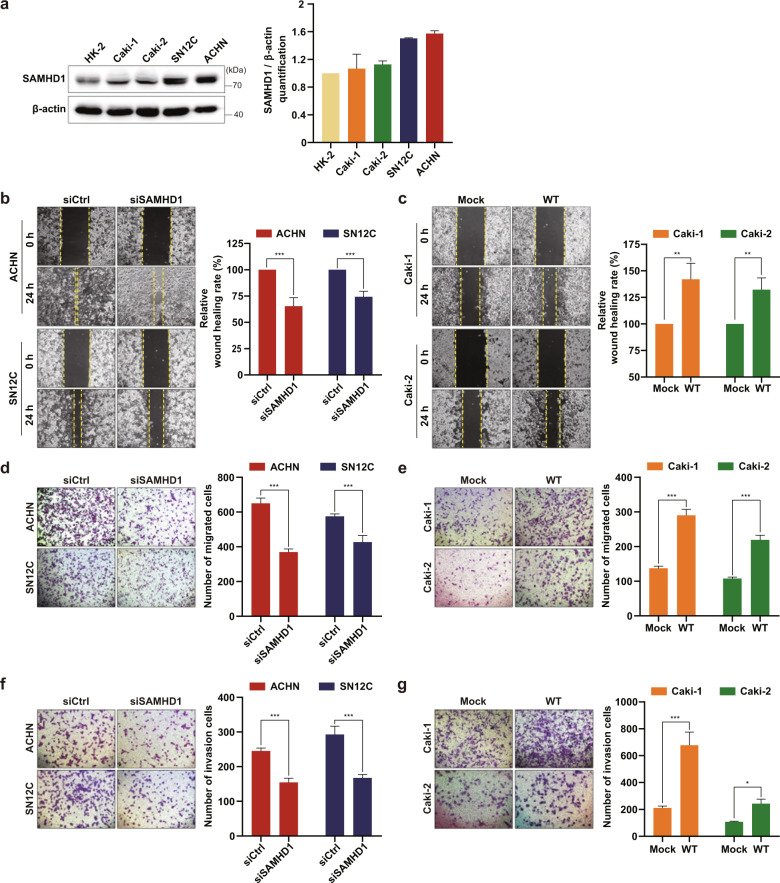
SAMHD1 accelerates the migration and invasion of human ccRCC cells. **a** Western blot analysis of SAMHD1 expression in normal renal epithelial cells (HK-2) and different RCC cell lines (Caki-1, Caki-2, SN12C, ACHN). Densitometric analysis of SAMHD1 protein expression levels relative to β-actin levels. **b, c** Wound-healing analysis of cell migration ability in SAMHD1-knockdown ACHN and SN12C cells or SAMHD1-overexpressed Caki-1 and Caki-2 cells over 24 h. Data are presented as the mean ± SD. The data shown are representative of three independent experiments. **p < 0.01; ***p < 0.001. **d, e** Transwell migration assay of cell migration capacity upon SAMHD1 knockdown in ACHN and SN12C cells or overexpression in Caki-1 and Caki-2 cells over 24 h. Data are presented as the mean ± SD. The data shown are representative of three independent experiments. ***p < 0.001. **f, g** Analysis of cell invasion ability upon SAMHD1 knockdown in ACHN and SN12C cells or overexpression in Caki-1 and Caki-2 cells. *p < 0.05; ***p < 0.001. Data are presented as the mean ± SD of three independent experiments.

To study the role of SAMHD1 in ccRCC, we first assessed the basal level of SAMHD1 expression in four ccRCC cell lines compared with that in the normal kidney epithelial cell line HK-2 (Fig. 2a). Based on the basal level of SAMHD1 in each cell line, Caki-1 and Caki-2 cells were used for SAMHD1 overexpression, and SN12C and ACHN cells were used for knockdown experiments. Analysis of cell proliferation ability indicated that SAMHD1 expression did not influence proliferation (Supplementary Fig. 2a, b). Subsequently, we examined the effect of SAMHD1 on cell migration using in vitro wound-healing assays and found that SAMHD1 knockdown suppressed cell migration in both the ACHN and SN12C cell lines (Fig. 2b), whereas SAMHD1 overexpression significantly promoted the migration of ccRCC cells (Fig. 2c). The transwell migration assay also showed consistent results (Fig. 2d, e). The invasion assay confirmed that SAMHD1 knockdown markedly inhibited the invasiveness of ACHN and SN12C cells (Fig. 2f), whereas exogenous SAMHD1 overexpression facilitated the invasiveness of Caki-1 and Caki-2 cells compared with control cells (Fig. 2g). These results indicate that SAMHD1 facilitates ccRCC cell migration.

### SAMHD1 enhances ccRCC cell mesenchymal characteristics to increase migration capacity

**Fig. 3:**
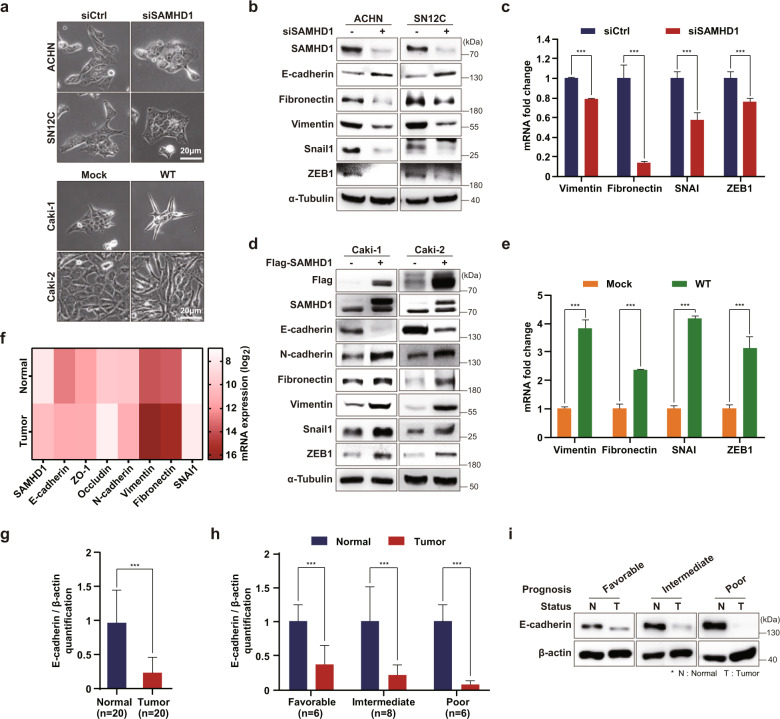
SAMHD1 enhances epithelial–mesenchymal transition. **a** Phase-contrast microscopy revealed changes in cell morphology from epithelial to mesenchymal characteristics in different ccRCC cells after SAMHD1 knockdown or overexpression. Scale bar, 20 μm. **b** Western blot analysis of epithelial and mesenchymal gene expression after SAMHD1 knockdown in ACHN and SN12C cells. **c** qPCR analysis of mesenchymal markers after SAMHD1 knockdown in SN12C cells. Data are presented as the mean ± SD. The experiments were independently performed at least three times. ***p < 0.001. **d** Western blot analysis of epithelial and mesenchymal markers in SAMHD1-overexpressing Caki-1 and Caki-2 cells. **e** qPCR analysis of mesenchymal markers in SAMHD1-overexpressed Caki-1 cells. Data are presented as the mean ± SD. The data shown are representative of three independent experiments. ***p < 0.001. **f** Association between the expression of SAMHD1 and that of epithelial or mesenchymal markers based on ccRCC clinical data. **g** E-cadherin protein levels relative to β-actin levels for 20 paired tumor and normal tissues. Data are presented as the mean ± SD. ***p < 0.001. **h** E-cadherin protein levels relative to β-actin levels in tissues of patients with ccRCC according to prognosis. Data are presented as the mean ± SD. **p < 0.01; ***p < 0.001. **i** Western blot analysis of E-cadherin expression in 20 paired tissue samples from patients with ccRCC. Western blot bands are presented with the corresponding prognosis. Data are presented as the mean ± SD.

Interestingly, we noted SAMHD1-induced changes in cell morphology that promoted cell migration or invasion depending on the SAMHD1 level. ACHN and SN12C cells could not form tips at their edges of SAMHD1 knockdown cells (Fig. 3a); conversely, when SAMHD1 was overexpressed in Caki-1 and Caki-2 cells, the cell body became pointed and appeared to be directional. Based on these findings, we hypothesized that SAMHD1 regulates cell migration through epithelial–mesenchymal transition, a well-known mechanism by which cancer cells acquire mesenchymal characteristics and increase motility by losing cell–cell junctions and basal polarity^37^. SAMHD1 knockdown upregulated the expression of E-cadherin—an epithelial marker—and downregulated that of mesenchymal markers, such as fibronectin, vimentin, snail1, and ZEB1 (Fig. 3b, c). In contrast, the opposite effects were observed following SAMHD1 overexpression (Fig. 3d, e). Furthermore, mRNA analysis of TCGA datasets showed consistent results with those obtained from western blotting and quantitative real-time PCR experiments (Fig. 3f). Similar to the in vitro results, E-cadherin expression was downregulated in tumor tissues compared to normal tissues of patients with ccRCC (Fig. 3g–i). Overall, these results suggest that SAMHD1 promotes EMT and enhances the mesenchymal characteristics required for effective ccRCC cell migration.

### SAMHD1 promotes the formation of lamellipodia in ccRCC cells

**Fig. 4:**
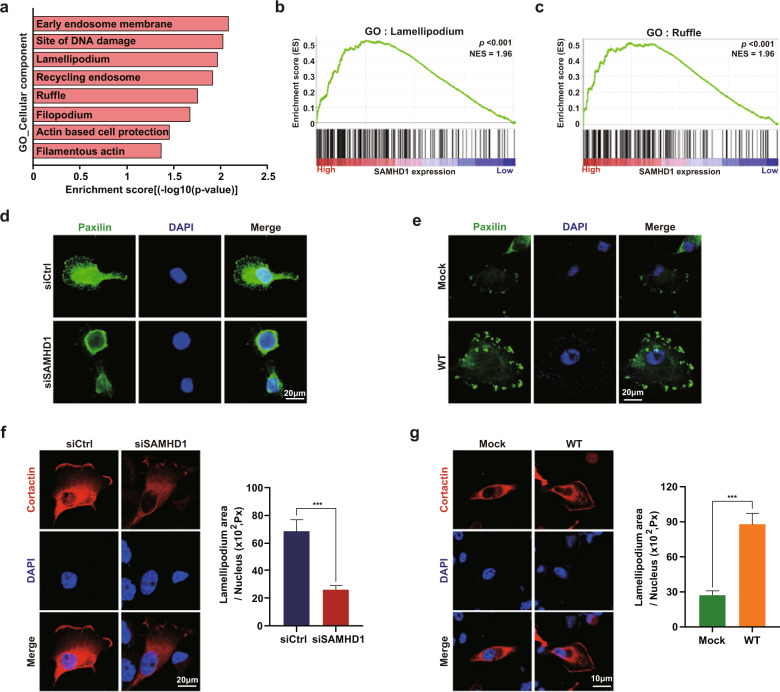
SAMHD1 is positively correlated with dynamic cytoskeletal changes in lamellipodia formation. **a** Gene Ontology (GO) analysis of the cellular component subcategories positively regulated by SAMHD1 overexpression. **b, c** Gene set enrichment analysis (GSEA) related to cytoskeletal changes for cell migration in the SAMHD1 high expression group. ***p < 0.001. **d, e** Immunofluorescence (IF) with anti-paxillin antibodies and 4′,6-diamidino-2-phenylindole (DAPI) staining of SAMHD1 knockdown SN12C cells or SAMHD1 overexpressed Caki-1 cells. Scale bar, 20 μm. **f** mCherry-cortactin was transfected as a control, and SAMHD1 was knocked down in SN12C cells for lamellipodium observation (left). Scale bar, 20 μm. Graphs present the lamellipodium area in each cell (right) (*n* = 20 cells). Data are presented as the mean ± SEM. ***p < 0.001. **g** SAMHD1-overexpressing Caki-1 cells and control cells were transfected with mCherry-cortactin for lamellipodium observation (left). Scale bar, 10 μm. Graphs present the lamellipodium area in each cell (right) (*n* = 20 cells). Data are presented as the mean ± SEM. ***p < 0.001.

Given that SAMHD1 acts as an oncogene and accelerates ccRCC cell migration, we hypothesized that SAMHD1 could positively regulate cell migration. Therefore, GSEA was performed using the ccRCC dataset from TCGA (dataset was downloaded through https://gdac.broadinstitute.org) to elucidate the detailed mechanism by which SAMHD1 regulates cell migration. The enriched GO subcategories indicated that the genes upregulated upon SAMHD1 overexpression were mainly associated with the structural features of actively migrating cells (Fig. 4a). Furthermore, we found that lamellipodia and membrane ruffles—subcategories in the cellular component category—were highly enriched (Fig. 4b, c). To further validate the GSEA results, we performed an immunofluorescence analysis of paxillin, which participates in nascent cell adhesion, spreading, and migration through interactions with multiple structural proteins^38^. Many paxillin-positive signals were observed at the cell periphery in the presence of SAMHD1 (Fig. 4d); however, paxillin-positive signals were barely present in SAMHD1-knockdown SN12C cells. Consistently, more paxillin-positive signals were observed in SAMHD1-overexpressing Caki-1 cells than in control cells (Fig. 4e). To assess the impact of SAMHD1 on lamellipodium or membrane ruffle development, ccRCC cells were transfected with RFP-cortactin, a well-known lamellipodium marker. As shown in Fig. 4f, the lamellipodium area was significantly reduced in SAMHD1-deficient cells compared with control cells. In contrast, SAMHD1-overexpressing Caki-1 cells had a larger lamellipodia area and stronger cortactin signals than those in control cells (Fig. 4g). Our findings suggest that SAMHD1 promotes lamellipodia formation during the nascent stage of cell migration.

### SAMHD1 increases small GTPase activities, especially Rac1 activity

**Fig. 5:**
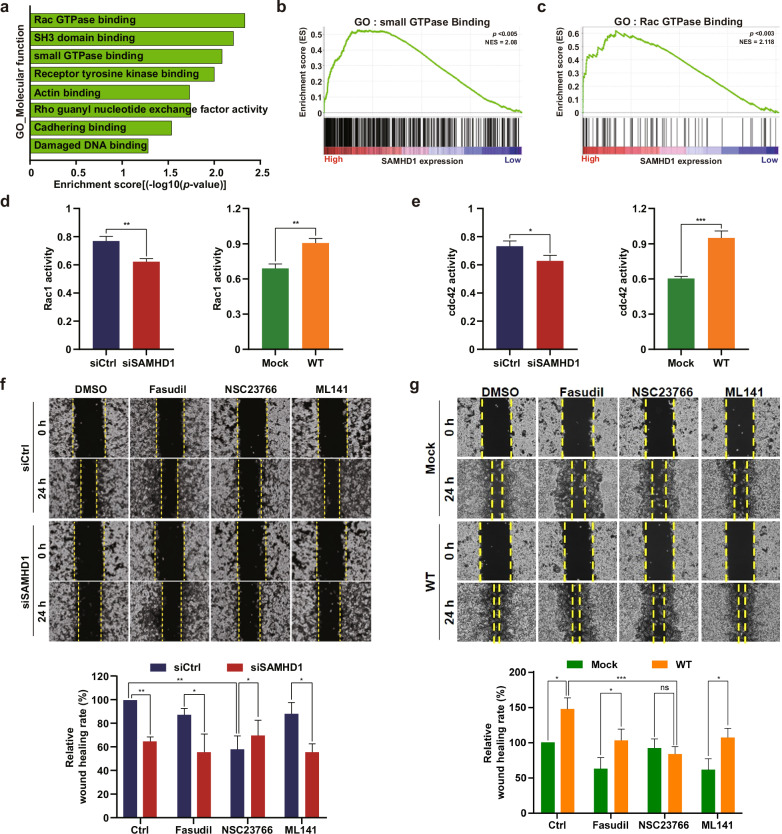
SAMHD1 regulates Rac1 activity and enhances ccRCC cell migration. **a** GO analysis was performed, and subcategories in the molecular function category were highly correlated with the high expression of SAMHD1. **b, c** Subcategories of the molecular function category related to cell migration. **p < 0.01. **d** A G-LISA-based assay was performed to measure GTP-bound Rac1 activity in SAMHD1-knockdown SN12C cells and SAMHD1-overexpressing Caki-1 cells. Data are presented as the mean ± SD. The data shown are representative of three independent experiments. **p < 0.01. **e** GTP-bound-cdc42 activity in SAMHD1-knockdown SN12C cells and SAMHD1-overexpressed Caki-1 cells. Data are presented as the mean ± SD. The experiments were independently performed three times. *p < 0.05; ***p < 0.001. **f** Wound-healing assay of cell migration ability with FAK inhibitor (PF573228), Rho A inhibitor (fasudil), Rac1 inhibitor (NSC23766), and cdc42 inhibitor (ML141). SAMHD1-knockdown SN12C cells were treated with each corresponding inhibitor at 10 μM over 24 h. The relative wound-healing area was normalized to that of the controls. Data are presented as the mean ± SD. The data shown are representative of three independent experiments. ***p < 0.001. **g** A wound healing assay was performed as in (**f**) in SAMHD1-overexpressing Caki-1 cells. The relative wound-healing area was normalized to that of DMSO-treated controls. *p < 0.05; ***p < 0.001. Data are presented as the mean ± SD of three independent experiments.

To further investigate the molecular mechanism that regulates the SAMHD1-mediated migration of ccRCC cells, we analyzed the molecular function category from the GSEA and found that the subcategories were mainly related to proteins regulating cell migration (Fig. 5a). In particular, the subcategories of small GTPase protein binding and Rac GTPase binding, which are essential for initiating lamellipodia formation, had high enrichment scores (Fig. 5b, c). To examine the causality between small GTPase activities and SAMHD1, Rac1 and cdc42 activities were measured following SAMHD1 knockdown or overexpression. Rac1 activity was decreased by SAMHD1 knockdown; however, increasing levels of exogenous SAMHD1 increased Rac1 activity to a similar level as that in the control (Fig. 5d). The activation of Rac1 and cdc42 in the early stages of cell migration is well established^39^. Consistent with the findings in previous reports, cdc42 activity with SAMHD1 knockdown and overexpression showed similar patterns as Rac1 activity (Fig. 5e). Rho A activity was also measured, but no significant difference in activity was observed (data not shown). Furthermore, to determine whether SAMHD1-mediated ccRCC cell migration is dependent on GTPase activation, a wound-healing assay was performed using small GTPase inhibitor treatments. Among the small GTPase inhibitors, the Rac1 inhibitor inhibited ccRCC cell migration to the same level as SAMHD1 knockdown (Fig. 5f). Similar results were observed in cells overexpressing SAMHD1, the ability of SAMHD1 to promote cell migration was completely blocked by the Rac1 inhibitor, NSC23766 (Fig. 5g). Our results suggest that Rac1 activation is essential for SAMHD1-induced cell migration.

### SAMHD1 binds with cortactin to promote endocytosis

**Fig. 6:**
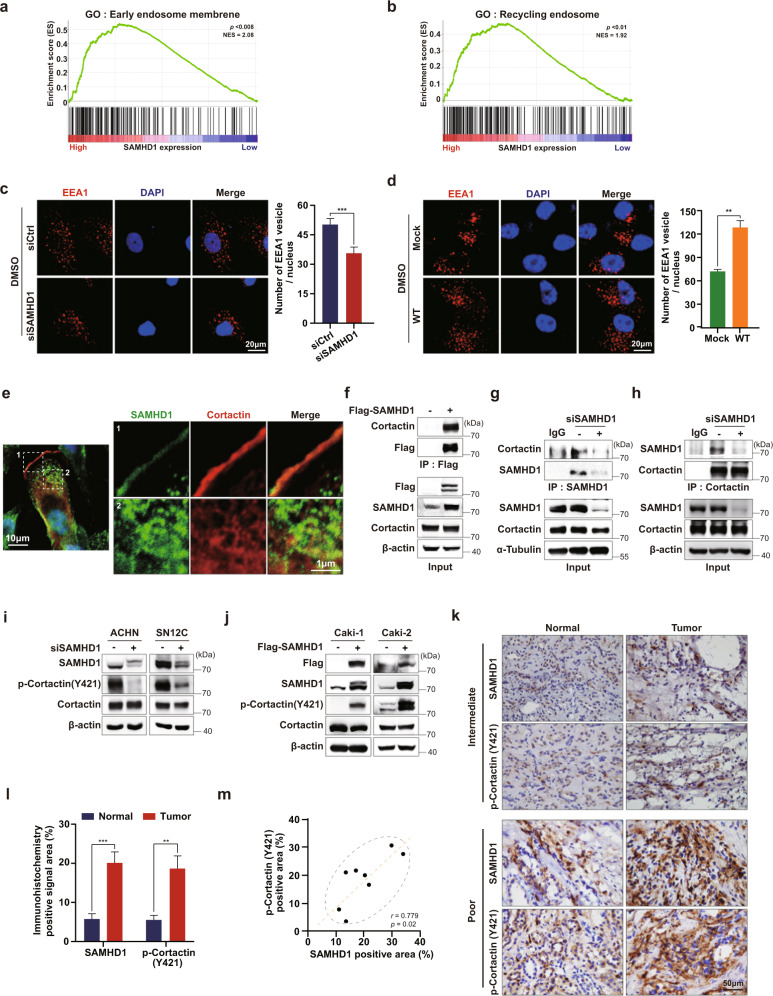
SAMHD1 participates in endosome formation and binds to cortactin directly. **a, b** GSEA analysis of *SAMHD1* mRNA levels and endocytosis-related signaling pathways. **c** ACHN cells were transfected with control or siSAMHD1 and immunolabeled for EEA1. The nuclei were stained with DAPI. Scale bar, 20 μm. Graphs present the number of EEA1-expressing vesicles per single cell (*n* = 55 cells). Data are presented as the mean ± SEM. The experiments were independently performed three times. ***p < 0.001. **d** SAMHD1-overexpressing Caki-1 cells were immunolabeled for EEA1. The number of EEA1-expressing vesicles per single cell is shown (*n* = 55 cells). Scale bar, 20 μm. Data are presented as the mean ± SEM. The data shown are representative of three independent experiments. ***p < 0.001. **e** SAMHD1 colocalized with cortactin in the cytoplasmic circular-shaped organelles and on the lamellipodia. **f** Exogenous Flag-tagged SAMHD1 was immunoprecipitated with magnetic Flag beads from Flag-SAMHD1-overexpressing Caki-1 cells. **g** Endogenous SAMHD1 was immunoprecipitated from SAMHD1-knockdown SN12C cells. Anti-cortactin antibodies were used to examine SAMHD1 and cortactin binding. IgGs were incubated with cell lysates as negative controls. **h** Endogenous cortactin was immunoprecipitated from SAMHD1-downregulated ACHN cells. Anti-SAMHD1 antibody was used to examine SAMHD1 and cortactin binding. IgG was incubated with cell lysates as a negative control. **i, j** Phosphorylated cortactin (Y421) and total cortactin levels were evaluated using western blotting in SAMHD1-knockdown ACHN and SN12C cells or SAMHD1-overexpressed Caki-1 and Caki-2 cells. **k** IHC analysis of SAMHD1 and phosphorylated cortactin (Y421) in ccRCC patient tissues. Scale bar, 50 μm. **l** Areas with positive IHC staining were measured and are shown on the graph. Data are presented as the mean ± SD. ***p < 0.001; **p < 0.01. **m** Correlation between SAMHD1 and phosphorylated cortactin (Y421) expression from IHC analysis of ccRCC patient tissues.

Endocytosis is an important recycling system during cancer progression, especially during metastasis. Cancer cells recycle cell-surface proteins for cell migration by internalizing selective vesicles. Hence, the faster endocytosis occurs, the faster metastasis proceeds^40,41^. Notably, GSEA revealed a relationship between SAMHD1 expression and early endosomes as well as recycling endosomes (Fig. 6a, b). Since the initiation of endocytosis stimulates Rac1 activation^42^, we hypothesized that endocytosis might be involved in the SAMHD1-mediated migration of ccRCC cells. To investigate whether the formation of intracellular vesicles is regulated by SAMHD1, the expression of EEA1, an early endosome marker, was analyzed using confocal microscopy. SAMHD1 knockdown reduced the total number of EEA1-expressing vesicles in SN12C cells (Fig. 6c). In contrast, overexpression of SAMHD1 increased the number of EEA1-expressing vesicles by two-fold compared to that in the Caki-1 control cells (Fig. 6d). These results indicate that SAMHD1 promotes endosome formation.

Consistent with previous reports, SAMHD1 was mainly expressed in the nucleus. However, SAMHD1 was observed in the cytoplasm and cell membrane to a lesser extent, but SAMHD1 localization in the cytoplasm and cell membrane was rarely found in SAMHD1-knockdown cells (Supplementary Fig. 3a). In addition, Flag-tagged exogenous SAMHD1 was found not only in the nucleus but also in the cytoplasm and cell membrane (Supplementary Fig. 3b). Interestingly, we observed that SAMHD1 colocalized with cortactin in the lamellipodia and cytoplasm (Fig. 6e). Based on this observation, we examined the interaction between SAMHD1 and cortactin using immunoprecipitation and observed exogenous and endogenous binding of SAMHD1 and cortactin (Fig. 6f–h). Tyrosine phosphorylation of cortactin is important for endosomal vesicle formation^27,43,44^. We found that the level of cortactin tyrosine phosphorylation was decreased in SAMHD1-depleted ACHN and SN12C cells (Fig. 6i). Conversely, SAMHD1 overexpression in Caki-1 and Caki-2 cells potentiated cortactin tyrosine phosphorylation levels (Fig. 6j). For further confirmation, we analyzed the correlation between SAMHD1 and cortactin phosphorylation in paired cancer and normal tissues from patients with ccRCC. Both SAMHD1 and phosphorylated cortactin levels were higher in cancer tissues than in adjacent normal tissues (Fig. 6k, l). The phosphorylation level of cortactin was positively correlated with the expression of SAMHD1 in tumor tissues (Fig. 6m).

**Fig. 7:**
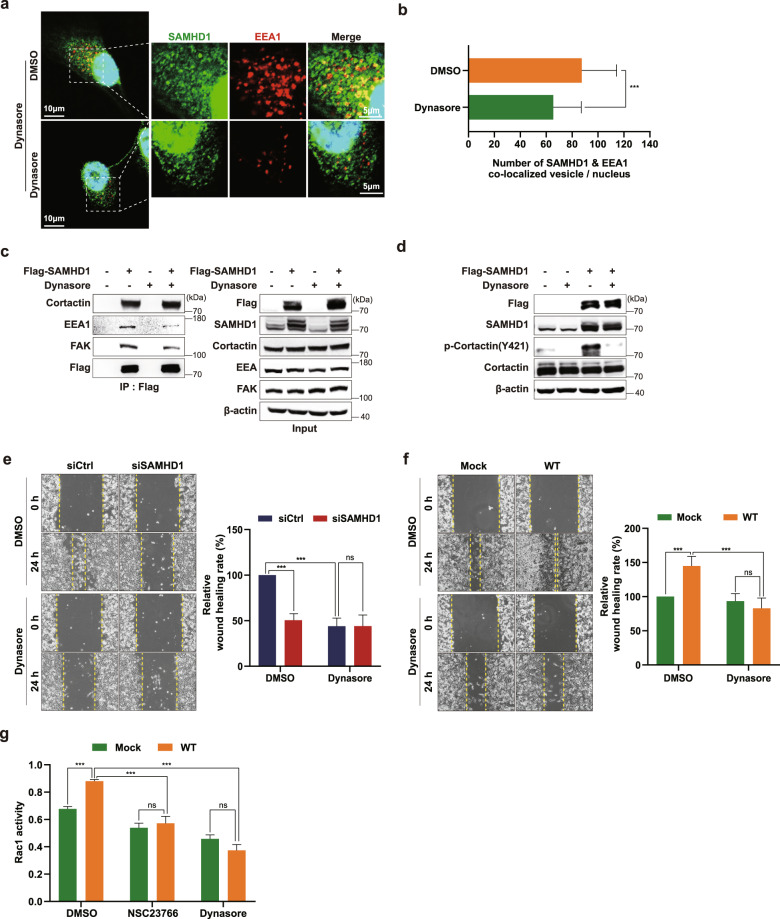
SAMHD1 contributes to endocytosis-dependent ccRCC cell migration. **a** IF staining with an anti-SAMHD1 antibody, an anti-EEA1 antibody, and DAPI in SAMHD1-overexpressed Caki-1 cells treated with 80 μM Dynasore over 24 h. **b** The number of EEA1 vesicles colocalized with SAMHD1 per cell was quantified and is shown on the graph (*n* = 30 cells). Data are presented as the mean ± SD. The data shown are representative of three independent experiments. ***p < 0.001. **c** IP assay was performed using Flag-tagged beads in Flag-SAMHD1-overexpressing Caki-1 cell lysates treated with 80 μM Dynasore over 24 h. **d** Phosphorylated cortactin (Y421) and total cortactin expression in SAMHD1-overexpressed Caki-1 cells treated with 80 μM Dynasore over 24 h and analyzed using western blotting. **e, f** Wound-healing assay of cell migration ability in SAMHD1-knockdown SN12C cells or in SAMHD1-overexpressed Caki-1 cells treated with 80 μM Dynasore. The relative wound-healing area was normalized to that of DMSO-treated controls. Data are presented as the mean ± SD. The data shown are representative of three independent experiments. ***p < 0.001. **g** G-LISA-based assay of GTP-bound Rac1 activity in SAMHD1-overexpressing Caki-1 cells treated with 10 μM NSC23766 or 80 μM Dynasore over 24 h. ***p < 0.001. Data are presented as the mean ± SD of three independent experiments.

To further determine the impact of endocytosis on SAMHD1-induced endosome formation, we treated cells with Dynasore, an endocytosis inhibitor. Dynasore treatment attenuated the colocalization of SAMHD1 and EEA1 in endosomal vesicles (Fig. 7a, b). In addition, the binding of the endosomal formation complex with SAMHD1 was markedly decreased by the inhibition of endocytosis (Fig. 7c). Dynasore also attenuated cortactin phosphorylation in SAMHD1-overexpressing cells (Fig. 7d), suggesting that cortactin phosphorylation is involved in SAMHD1-mediated endosomal complex formation.

Furthermore, to determine whether SAMHD1-mediated ccRCC cell migration is maintained by endocytosis, we performed a wound-healing assay with an endocytosis inhibitor. As shown in Fig. 7e, the movement of cells was slowed down with Dynasore treatment in control cells compared with SAMHD1 knockdown cells. Furthermore, the ability of SAMHD1 to induce cell migration was eliminated after Dynasore treatment (Fig. 7f). Based on accumulating evidence that endocytosis contributes to Rac1 activation^42,45,46^, we investigated whether SAMHD1-induced endocytosis could regulate Rac1 activation in Dynasore-treated cells. Dynasore treatment impaired SAMHD1-mediated Rac1 activation (Fig. 7g), suggesting that Rac1 is downstream of endocytosis signaling. Collectively, these findings suggest that SAMHD1-mediated endocytosis promotes ccRCC cell migration through Rac1 activation.

### SAMHD1 activates endosomal FAK signaling

**Fig. 8:**
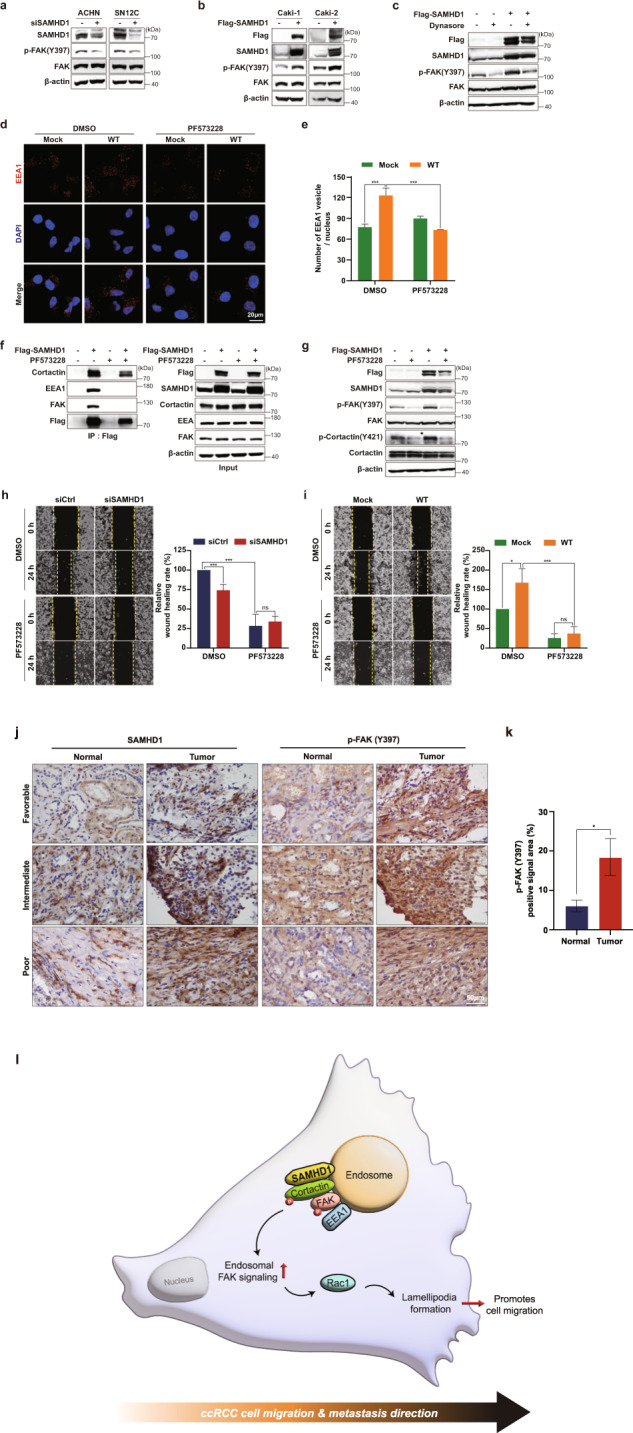
SAMHD1 activates endosomal FAK signaling. **a, b** Western blot analysis of phospho-FAK (Y397) and total FAK levels in SAMHD1-knockdown ACHN and SN12C cells or SAMHD1-overexpressed Caki-1 and Caki-2 cells. **c** Western blot analysis of phosphorylated FAK (Y397) and total FAK expression in SAMHD1-overexpressed Caki-1 cells treated with 80 μM Dynasore over 24 h. **d** SAMHD1-overexpressed Caki-1 cells were treated with 10 μM PF573228 for 24 h and immunolabeled for EEA1. The nuclei were stained with DAPI. Scale bar, 20 μm. **e** Graphs show the number of EEA1 vesicles per single cell (*n* = 55 cells). ***p < 0.001. **f** IP assay was performed using Flag-tagged beads in Flag-SAMHD1-overexpressing Caki-1 cell lysates treated with 10 μM PF573228 over 24 h. **g** Western blot analysis of phosphorylated FAK (Y397) and phosphorylated cortactin (Y421) expression in SAMHD1-overexpressed Caki-1 cells treated with 10 μM PF573228 over 24 h. **h, i** Wound-healing assay of cell migration ability in SAMHD1-knockdown SN12C cells or SAMHD1-overexpressed Caki-1 cells treated with 10 μM PF573228. The relative wound-healing area was normalized to that of the controls. Data are presented as the mean ± SEM. The experiments were performed at least three times. *p < 0.05, ***p < 0.001. **j** IHC of SAMHD1 and p-FAK (Y397) expression in tissues of patients with ccRCC. Scale bar, 50 μm. **k** Areas with positive IHC staining were measured and are presented in the graph. Data are presented as the mean ± SD. *p < 0.05. **l** SAMHD1 promotes ccRCC metastasis through endosomal FAK signaling activation.

FAK is a well-known binding partner of cortactin and participates in various cell migration processes, lamellipodia formation, and endocytosis. Autophosphorylation of FAK at Y397 is necessary for cortactin phosphorylation and Rac1 activation^22,47,48^. Accordingly, we examined whether FAK phosphorylation was regulated by SAMHD1. FAK phosphorylation levels at Y397 decreased in SAMHD1*-*knockdown cells (Fig. 8a); in contrast, SAMHD1 overexpression increased FAK phosphorylation levels (Fig. 8b). Dynasore treatment impaired SAMHD1-induced FAK phosphorylation, indicating that SAMHD1-mediated endocytosis is required for FAK phosphorylation (Fig. 8c). To determine whether FAK participates in SAMHD1-induced endosome formation, we treated cells with the selective FAK inhibitor PF573228. In the presence of PF573228, SAMHD1-overexpressing cells did not form EEA1-expressing vesicles (Fig. 8d, e). In addition, treatment with PF573228 inhibited the binding of endosomal components to SAMHD1 (Fig. 8f). Consistent with endosomal complex formation, SAMHD1-induced cortactin phosphorylation was also diminished by FAK inhibitor treatment (Fig. 8g). To investigate whether FAK signaling is essential for SAMHD1-mediated ccRCC cell migration, we performed a wound-healing assay with FAK inhibitor treatment. PF573228 treatment reduced the motility of control cells to a level similar to that of SAMHD1-deficient cells (Fig. 8h). In addition, the effect of SAMHD1 overexpression on cell migration was reduced by PF573228 treatment (Fig. 8i). Collectively, our results suggest that SAMHD1-induced endosomal FAK signaling promotes ccRCC cell migration.

Finally, we analyzed FAK activation in tumor tissues from patients with ccRCC. Consistent with the pattern of SAMHD1 expression, FAK phosphorylation levels were higher in cancer tissues than in normal tissues (Fig. 8j, k). These findings confirm the important role of SAMHD1-promoted FAK phosphorylation in mediating ccRCC development.

## Discussion

Every year, 403 000 new cases of renal cell carcinoma are diagnosed worldwide. Most patients with renal cancer only become aware of symptoms after the cancer has progressed to a later stage, with only 10% of patients reporting symptoms in the early stages of the disease. Therefore, 33% of patients are diagnosed with stage IV metastatic renal cell carcinoma, which has a 5-year survival rate of only 12%^49,50^. Despite medical and therapeutic advances, effective strategies to prevent renal cancer metastasis are limited; therefore, insights into the molecular mechanisms promoting kidney cancer metastasis are urgently needed.

In the present study, we identified SAMHD1 as an oncogene in ccRCC and proposed a novel mechanism in cell migration. We found that SAMHD1-mediated endocytic FAK signaling promotes lamellipodia formation in ccRCC cells (Fig. 8l), SAMHD1 binds to cortactin to promote early endosome formation and stimulates Rac1 activation via FAK autophosphorylation, and activated Rac1 promotes lamellipodia protrusion, which initiates ccRCC cell migration.

The function of SAMHD1 in cancer is unclear; whether SAMHD1 plays the role of an oncogene or cancer suppressor remains controversial. We assumed that SAMHD1 might function through multiple pathways in different types of cancer. *Wu* et al. ^51^ reported that SAMHD1 acts as a tumor suppressor through the STING pathway during cancer progression in lung adenocarcinoma. TCGA analysis showed that *SAMHD1* mRNA levels were much lower in tumor tissues than in nontumor tissues in several cancer types, such as colorectal adenocarcinoma, bladder urothelial carcinoma, lung adenocarcinoma, and lung squamous cell carcinoma. In contrast, other types of cancers, such as glioma, uterine corpus endometrial carcinoma, and especially pankidney cancer, had much greater *SAMHD1* mRNA levels in tumor tissues than in nontumor tissues (Fig. 1a), indicating that SAMHD1 serves as a tumor suppressor or oncogene depending on the type of cancer. Herein, we provide strong evidence for the oncogenic roles of SAMHD1 in ccRCC supported by various in vitro assays together with ccRCC patient sample analysis. Our findings suggest a new clinical approach with SAMHD1 as a therapeutic marker for ccRCC treatment.

SAMHD1 expression was significantly increased upregulated in cancer tissues compared to normal tissues in RCC among several cancer types. The overexpression of SAMHD1 in RCC tumor tissues was also related to worse survival prognoses in groups with high SAMHD1 expression, especially for patients with the ccRCC subtype. Although we found no significant differences in survival between high and low SAMHD1-expression groups in PRCC and chRCC, some patients expressing high SAMHD1 showed better survival rates than those of patients expressing low SAMHD1, although this finding may be an artifact of small sample size^52^. Only 20% of RCC patients are diagnosed with the PRCC and chRCC subtypes^49^; therefore, less information is available for PRCC and chRCC than for ccRCC. This is especially the case for chRCC datasets, which lack the appropriate sample size and longitudinal data for survival analysis.

SAMHD1 is mainly observed in the nucleus and is thus widely considered to be a nuclear protein^8,53^; however, SAMHD1 has also been detected in the cytoplasm. Recent reports have indicated the importance of nucleocytoplasmic shuttling to the function of SAMHD1. SAMHD1 can translocate into the cytoplasm through stimulation by growth factors, and shifting its localization to the cytoplasm is required for LINE-1 inverse enzyme inhibition^9,54^. In contrast to studies stating that SAMHD1 only has catalytic activity as a deoxyribonucleoside triphosphohydrolase in the nucleus^8,53^, the restriction activity of HIV-I was confirmed in an NLS mutant of SAMHD1 or with its NLS inactivated cells, suggesting a cytoplasmic function for SAMHD1. In this study, we confirmed that SAMHD1 was localized in both the cytoplasm and cell membrane in addition to the nucleus. Furthermore, we found that SAMHD1 participates in lamellipodia formation and endocytosis by binding to cortactin. This result is consistent with the findings of a previous study that demonstrated that SAMHD1 binds to the transmembrane protein CD81^55^. The function of SAMHD1 is regulated via various posttranslational modifications, such as phosphorylation, acetylation, methylation, and SUMOylation^16,56–59^. In particular, SAMHD1 phosphorylation at T592 is important for determining its localization in the cytoplasm^60^. Therefore, we investigated whether the level of SAMHD1 phosphorylated at T592 affected patient prognosis but found no significant difference in the phosphorylation level of SAMHD1 normalized to total SAMHD1 between the normal and tumor tissues of ccRCC patients under various pathological conditions (data not shown). Although SAMHD1 phosphorylation at T592 is not involved in regulating the malignancy of ccRCC, further studies are required to examine whether the ability of SAMHD1 to induce endocytosis and lamellipodia formation are regulated by other posttranslational modifications.

We found that SAMHD1 colocalized with EEA1, which led to the formation of early endosomes to facilitate cell migration (Fig. 7a). Consistent with our findings, *Rocha-Perugini* et al. ^55^ reported that cytoplasmic vesicles generated by SAMHD1 are surrounded by EEA1, which protects them from proteasomal degradation. They also showed that vesicles containing SAMHD1 were unrelated to late endosomes, multivesicular bodies (CD63), or lysosomes. *Yuan* et al. ^61^ also showed that SAMHD1 was a predicted binding partner of Rab21 in Rab21-mediated vesicle trafficking using mass spectrometry. Increasing evidence suggests that Rab21 is involved in early endosome formation and is constantly recycled, together with vesicles, to the cell membrane to promote cell migration rather than degradation^31,62–64^. Based on these previous findings, our study provides new insights into the regulatory mechanisms of SAMHD1-mediated early endosome formation.

Cortactin is an actin-binding protein that interacts with a wide range of proteins and participates in lamellipodia development in cell adhesion and migration^23,24,27^. We identified SAMHD1 as a novel binding partner of cortactin and confirmed that they were colocalized in the plasma membrane and endosomes (Fig. 6e). Although the exact binding site of these proteins is unclear, GSEA analysis suggests that SAMHD1 binds to the SH3 domain, one of three major cortactin domains, with a high probability (Fig. 5a). It is likely that SAMHD1 binds to the SH3 domain of cortactin as this domain is where other binding partners interact with cortactin. Further studies are needed to determine the cortactin binding domain that SAMHD1 interacts with.

Overall, our study presents a clearly elucidated pathway by which SAMHD1 regulates endosomal FAK signaling. In this cascade signaling axis, we identified cortactin as a novel binding partner of SAMHD1 that participates in endocytosis and lamellipodia formation. Furthermore, activated FAK signaling stimulates Rac1 activity, inducing lamellipodia protrusion for ccRCC cell migration. In conclusion, the SAMHD1-mediated endosomal FAK signaling axis could serve as a novel therapeutic target for ccRCC.

## Supplementary information

Supplementary Figure
