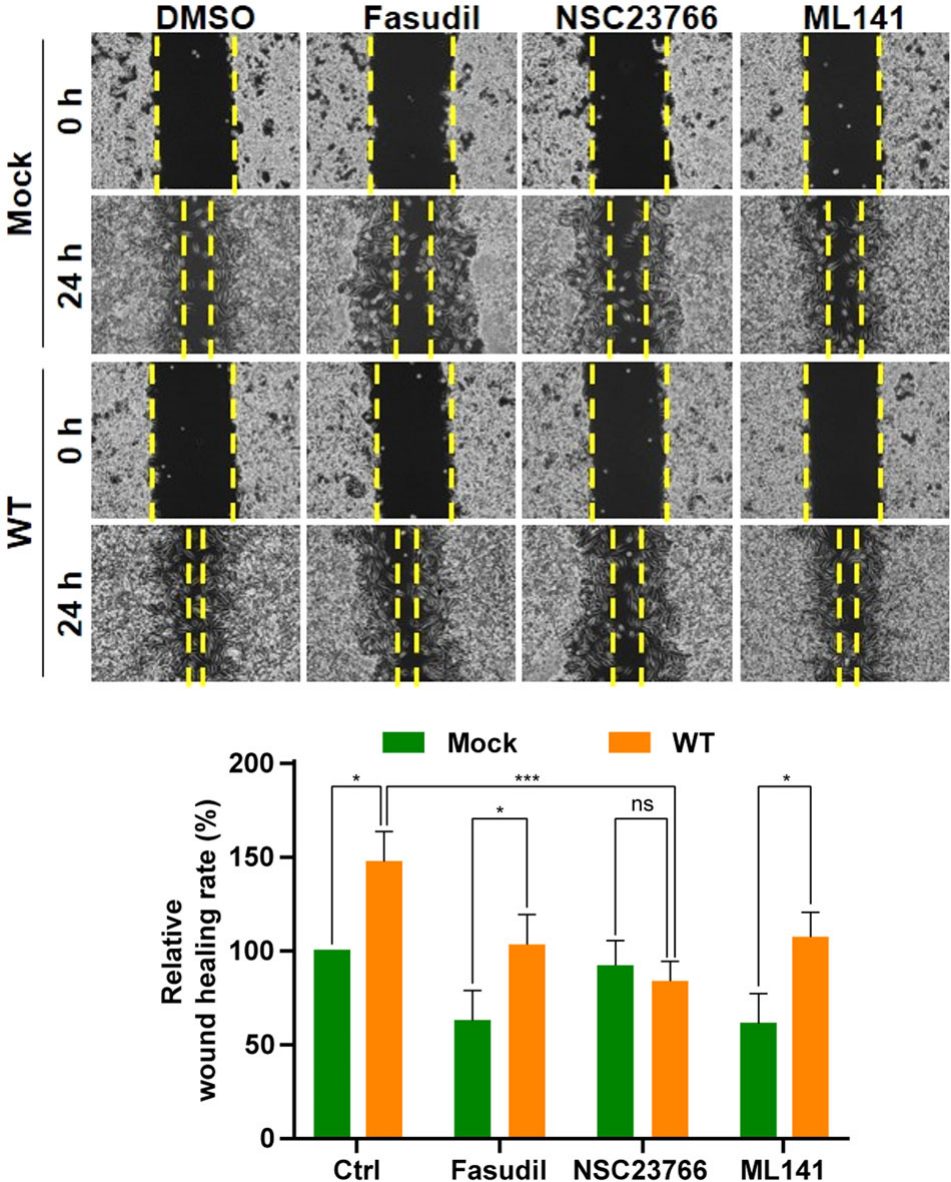# Author Correction: SAMHD1-induced endosomal FAK signaling promotes human renal clear cell carcinoma metastasis by activating Rac1-mediated lamellipodia protrusion

**DOI:** 10.1038/s12276-025-01502-4

**Published:** 2025-07-29

**Authors:** Sunho An, Tam Thuy Lu Vo, Taekwon Son, Hoon Choi, Jinyoung Kim, Juyeon Lee, Byung Hoon Kim, Misun Choe, Eunyoung Ha, Young-Joon Surh, Kyu-Won Kim, Ji Hae Seo

**Affiliations:** 1 College of Pharmacy and Research Institute of Pharmaceutical Sciences, Seoul National University, Seoul, 08826 South Korea; 2 Department of Biochemistry, School of Medicine, Keimyung University, Daegu, 42601 Republic of Korea; 3 Korea Brain Bank, Korea Brain Research Institute, Daegu, 42601 Republic of Korea; 4 Department of Internal Medicine, School of Medicine, Keimyung University, Daegu, 42601 Republic of Korea; 5 Department of Urology, School of Medicine, Keimyung University, Daegu, 42601 Republic of Korea; 6 Department of Pathology, School of Medicine, Keimyung University, Daegu, 42601 Republic of Korea

**Keywords:** Lamellipodia, Endocytosis, Renal cell carcinoma, Oncogenes, RHO signalling

Correction to: *Experimental & Molecular Medicine* 10.1038/s12276-023-00961-x, published online 03 April 2023

After online publication of this article, the authors noticed an error in the Fig. 5g section.

The correct statement of this article should have read as below.

In Fig. 5g, the authors identified that the control (Mock) result (ML141 treated for 24 h) was inadvertently duplicated with the WT result (NSC23766 treated for 24 h) during the data organization process. To rectify this duplication, we prepared the correct version of this figure with the proper microscopy picture of control cells (mock) treated with ML141 for 24 h. Fig. 5g shows that SAMHD1-overexpressing Caki-1 cells treated with NSC23766, a Rac1 inhibitor, for 24 h exhibited a marked reduction in migration, comparable to control levels. In contrast, inhibitors targeting RhoA or Cdc42 had no significant impact on SAMHD1-mediated migration. This data suggest that Rac1 activation is essential for SAMHD1-induced cell migration. Thus, our conclusion about the crucial role of SAMHD1 overexpression in RCC cell migration is not influenced by the correction.

The authors apologize for any inconvenience caused.

**Original Figure 5g.** 
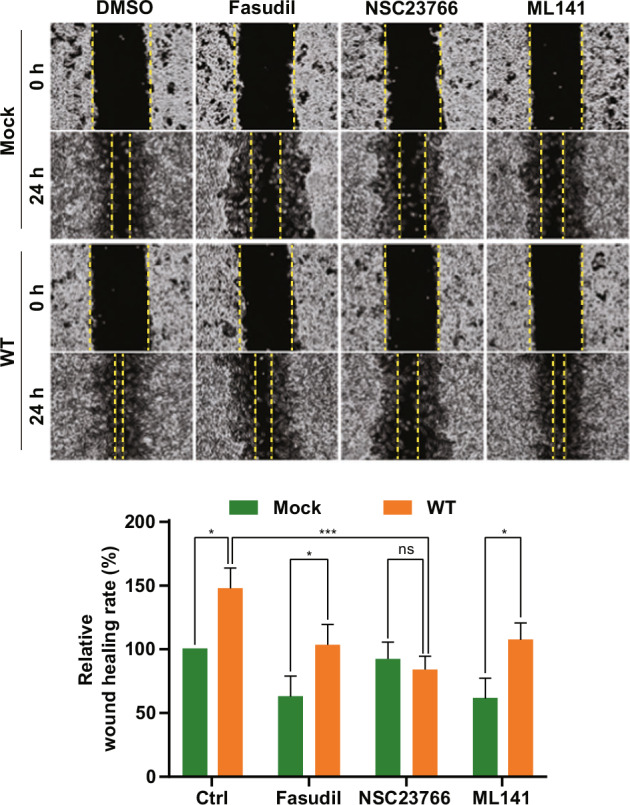


**Corrected Figure 5g.**