# Supplementary material for: SAMHD1-induced endosomal FAK signaling promotes human renal clear cell carcinoma metastasis by activating Rac1-mediated lamellipodia protrusion
Source: Exp Mol Med. 2023 Apr 3;55(4):779–93. doi: 10.1038/s12276-023-00961-x (PMC10167369; doi:10.1038/s12276-023-00961-x)
Supplement: Supplementary file 1 — Supplementary Figure [file 12276_2023_961_MOESM1_ESM.pdf]

**Supplementary 1.** *An et al.*

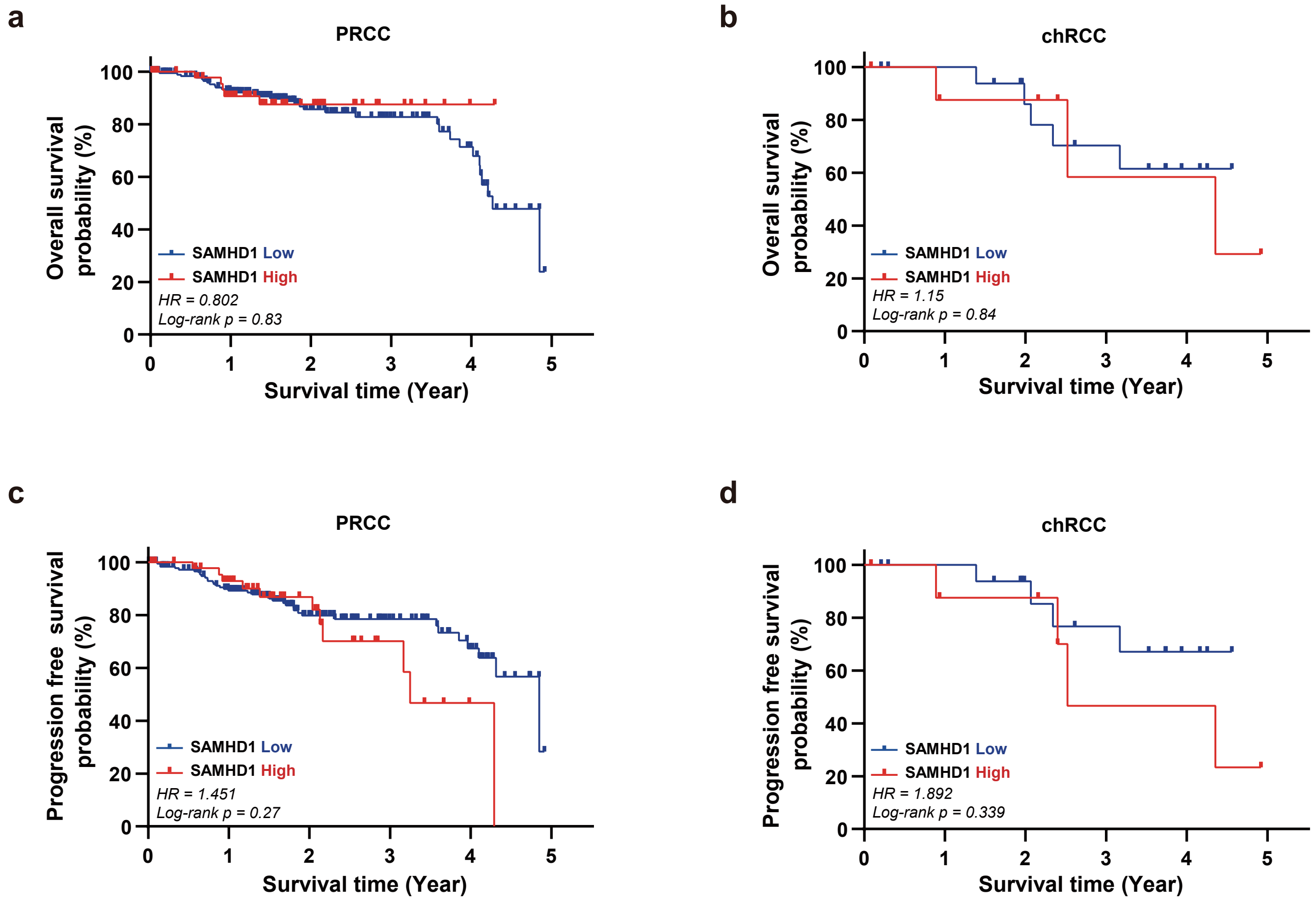

**Supplementary 2.** *An et al.*

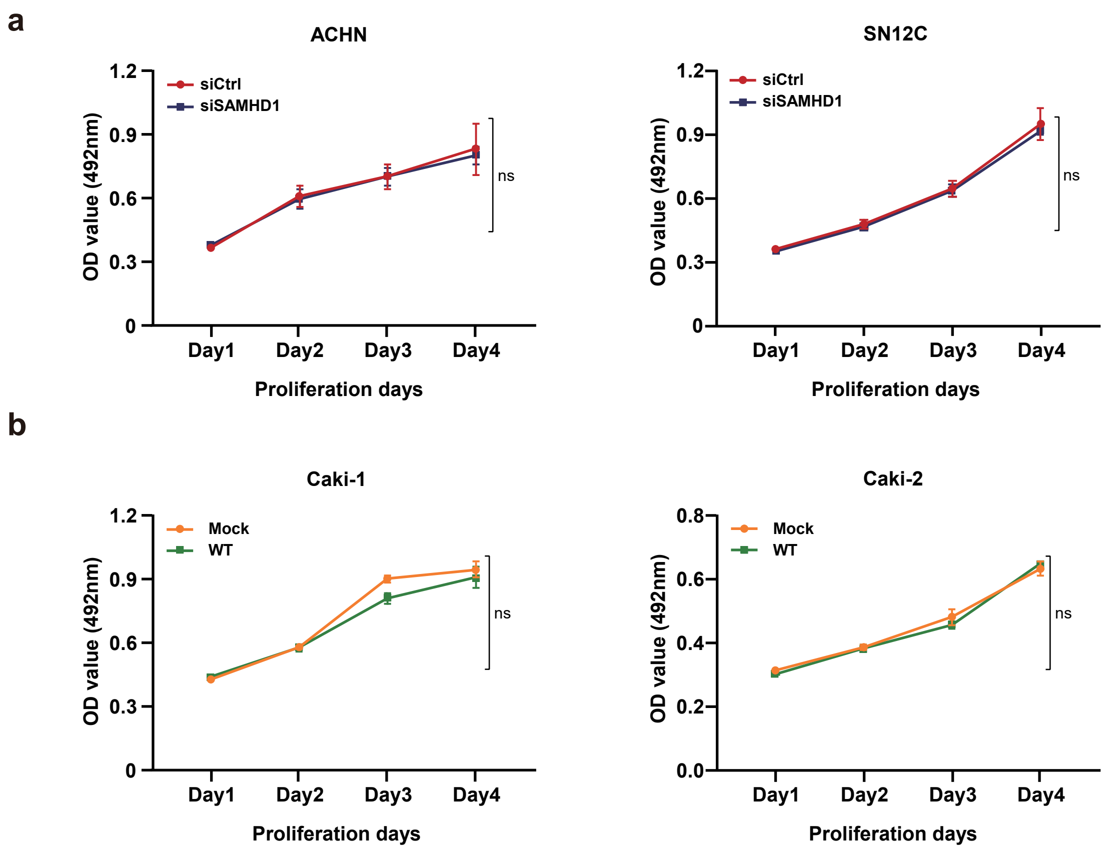

# Supplementary 3. *An et al.*

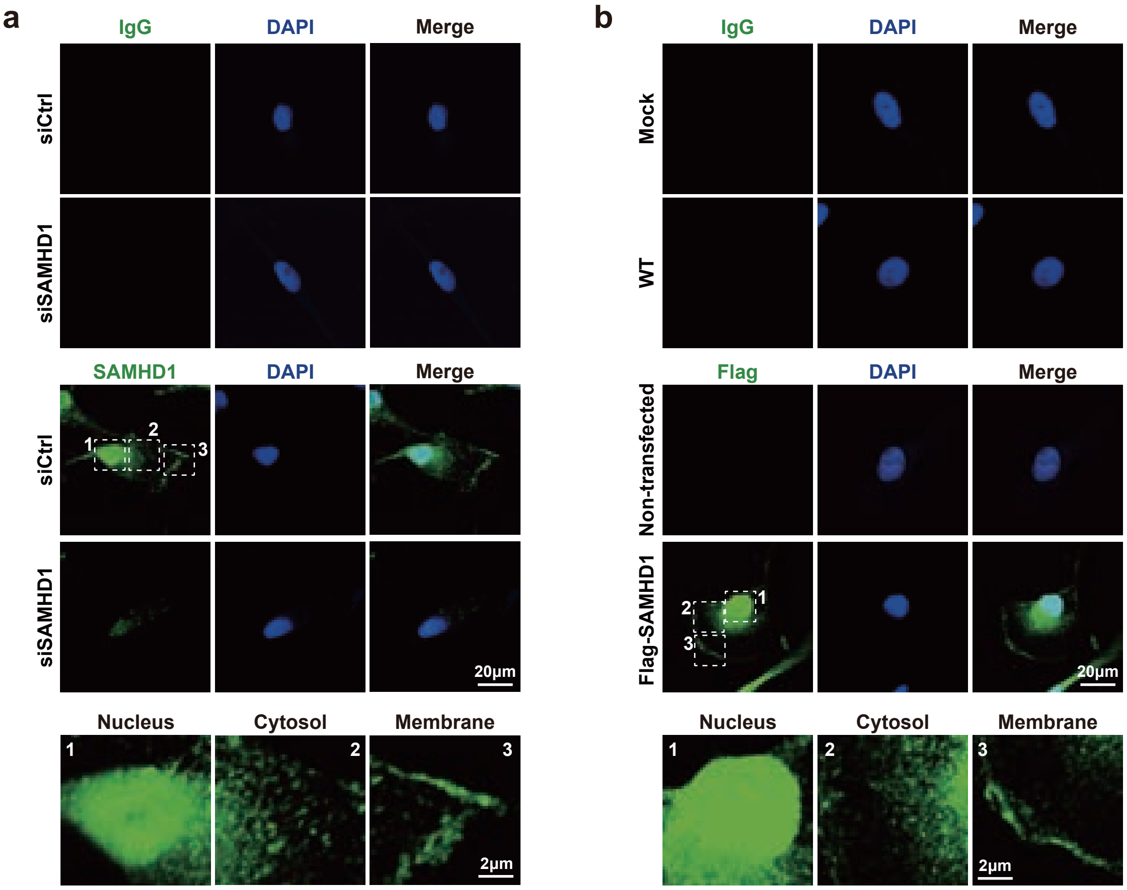

# Supplementary Information

**Supplementary Fig. S1. SAMHD1 does not affect patient survival rates in the renal papillary cell carcinoma (PRCC) and kidney chromophobe (chRCC) subtypes.**

**(a)** Kaplan–Meier analysis of 5-year overall survival (OS) between PRCC patient groups with high (n = 50) and low SAMHD1 expression (n = 187). Log-rank $p = 0.83$. **(b)** Kaplan–Meier analysis of 5-year OS between chRCC patient groups with high (n = 9) and low SAMHD1 expression (n = 18). Log-rank $p = 0.84$. **(c)** Kaplan–Meier analysis of 5-year progression-free survival (PFS) between PRCC patient groups with high (n = 49) and low SAMHD1 expression (n = 186). Log-rank $p = 0.27$. **(d)** Kaplan–Meier analysis of 5-year PFS between chRCC patient groups with high (n = 9) and low SAMHD1 expression (n = 18). Log-rank $p = 0.339$.

**Supplementary Fig. S2. SAMHD1 does not affect cell proliferation**

**(a, b)** Cell proliferation assay was performed using MTS reagent in SAMHD1-knockdown ACHN (left) and SN12C cells (right) **(a)** or SAMHD1-overexpressed Caki-1 (left) and Caki-2 cells (right) **(b)**. Data are presented as the mean ± SD of three independent experiments.

**Supplementary Fig. S3. SAMHD1 mainly localizes in the nucleus and partially in the cytoplasm and cell membrane.**

**(a)** IF with anti-IgG (negative control), anti-SAMHD1 antibodies, and 4′,6-diamidino-2-phenylindole (DAPI) staining of SAMHD1-knockdown Caki-1 cells. **(b)** IF staining with anti-

Flag antibody and DAPI of SAMHD1-overexpressed Caki-1 cells. Anti-IgG antibody was used as a negative control.
